# System‐level metabolic modeling facilitates unveiling metabolic signature in exceptional longevity

**DOI:** 10.1111/acel.13595

**Published:** 2022-03-27

**Authors:** Gong‐Hua Li, Feifei Han, Fu‐Hui Xiao, Kang‐Su‐Yun Gu, Qiu Shen, Weihong Xu, Wen‐Xing Li, Yan‐Li Wang, Bin Liang, Jing‐Fei Huang, Wenzhong Xiao, Qing‐Peng Kong

**Affiliations:** ^1^ State Key Laboratory of Genetic Resources and Evolution/Key Laboratory of Healthy Aging Research of Yunnan Province Kunming Institute of Zoology Chinese Academy of Sciences Kunming Yunnan China; ^2^ Kunming Key Laboratory of Healthy Aging Study Kunming Yunnan China; ^3^ 2348 Harvard Medical School Immune and Metabolic Computational Center Massachusetts General Hospital Boston Massachusetts USA; ^4^ 12635 School of Life Sciences Center for Life Sciences Yunnan University Kunming Yunnan China; ^5^ CAS Center for Excellence in Animal Evolution and Genetics Chinese Academy of Sciences Kunming Yunnan China; ^6^ KIZ/CUHK Joint Laboratory of Bioresources and Molecular Research in Common Diseases Kunming Yunnan China

**Keywords:** aging, GPMM, longevity, metabolic modeling, omics integration, systems biology

## Abstract

Although it is well known that metabolic control plays a crucial role in regulating the health span and life span of various organisms, little is known for the systems metabolic profile of centenarians, the paradigm of human healthy aging and longevity. Meanwhile, how to well characterize the system‐level metabolic states in an organism of interest remains to be a major challenge in systems metabolism research. To address this challenge and better understand the metabolic mechanisms of healthy aging, we developed a method of genome‐wide precision metabolic modeling (GPMM) which is able to quantitatively integrate transcriptome, proteome and kinetome data in predictive modeling of metabolic networks. Benchmarking analysis showed that GPMM successfully characterized metabolic reprogramming in the NCI‐60 cancer cell lines; it dramatically improved the performance of the modeling with an *R* ^2^ of 0.86 between the predicted and experimental measurements over the performance of existing methods. Using this approach, we examined the metabolic networks of a Chinese centenarian cohort and identified the elevated fatty acid oxidation (FAO) as the most significant metabolic feature in these long‐lived individuals. Evidence from serum metabolomics supports this observation. Given that FAO declines with normal aging and is impaired in many age‐related diseases, our study suggests that the elevated FAO has potential to be a novel signature of healthy aging of humans.

## Graphical abstract

Little is known for the systems metabolic profile of centenarians, the paradigm of human healthy aging and longevity. Here, we developed a method of genome‐wide precision metabolic modeling (GPMM) to systematically analyze human metabolic networks. We used GPMM to examine the metabolic networks of centenarian and demonstrated the elevated fatty acid oxidation as a novel signature of healthy aging of humans.

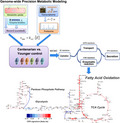

**Abbreviations**
CEN: centenarian
F1: centenarian‐children
F1SP: spouses of centenarian‐children
FAO: fatty acid oxidation
GPMM: genome‐wide precision metabolic modeling

## 1 INTRODUCTION

Population aging is an increasingly urgent issue confronting many countries worldwide (Chang et al., 2019). As most disabilities and fatal human diseases are age‐related (DALYs & Collaborators, 2018), understanding the mechanisms of aging will help with the development of therapeutics for aging‐related diseases. Metabolic control plays a crucial role in regulating the health span and life span of various organisms, for example, worms (Leiser et al., 2015) and primates (Mattison et al., 2012). Dysregulated metabolism often leads to premature aging and certain diseases in humans (López‐Otín et al., 2016). In contrast, long‐lived people, such as centenarians (CENs), may have “healthy” metabolic profiles that support them in resisting age‐ and metabolic‐related diseases, although the exact mechanism remains elusive (López‐Otín et al., 2016).

Human metabolism is a complex network that contains thousands of reactions and metabolites, and systematic identification of metabolic changes in health and diseases remains challenging (Brunk et al., 2018). Constraint‐based reconstruction and analysis (COBRA) is based on flux balance analysis theory (Orth et al., 2010) and uses different types of constraints, including metabolite availability, nutrient limits, and the most widely available data, gene expression from either microarray or RNA‐seq, to build tissue‐specific metabolic models (Blazier & Papin, 2012; O’Brien et al., 2015; Vlassis et al., 2014; Wang et al., 2012) and perform metabolic modeling (Bordbar et al., 2014; Le Novere, 2015; Mardinoglu et al., 2014, 2014; Yizhak et al., 2015). Many COBRA methods have been developed to perform the metabolic modeling, and most of them were merged into the COBRA toolbox, a desktop software suite of interoperable COBRA methods (Becker et al., 2007; Heirendt et al., 2019; Schellenberger et al., 2011). COBRA methods have been widely used for modeling cellular metabolism (Bintener et al., 2020; Heirendt et al., 2019; Nam et al., 2012), and discovering disease mechanisms (Lewis et al., 2010; Mardinoglu et al., 2018), targets (Larsson et al., 2020; Mardinoglu et al., 2014), and drug candidates (Agren et al., 2014; Bintener et al., 2020).

A major challenge in previous metabolic modeling studies is the “low accuracy” in predicting metabolic fluxes in human cells, largely due to the fact that they considered merely qualitative data, rather than quantitative information. In the most commonly used methods (Agren et al., 2012; Blazier & Papin, 2012; Pacheco et al., 2019; Shlomi et al., 2008; Vlassis et al., 2014; Wang et al., 2012), quantitative gene expression or proteomics data need to be translated into qualitative values (O’Brien et al., 2015). Such kind of doing inevitably leads to the loss of most of the quantitative information and thus introduces biases in predicting metabolic fluxes in human cells.

In this work, we present a systems biology approach to quantitatively integrate omics (i.e., transcriptome and proteome) data and kinetome information into genome‐wide precision metabolic modeling (GPMM), aiming to accurately identify metabolic changes in human health and diseases. To benchmark its performance, we applied GPMM and other methods commonly used for metabolic modeling on the same transcriptome data from the NCI‐60 cell lines (Reinhold et al., 2012) to compare the predicted metabolic fluxes with the experimentally measured values. GPMM robustly and reliably predicted the experimentally measured fluxes and significantly outperformed the existing methods. We then applied GPMM to study the metabolism of a Chinese centenarian cohort to understand why CENs can delay or avoid many serious age‐related diseases. We found that elevated fatty acid oxidation (FAO) is the most significant metabolic feature in the CENs. Further serum metabolomic data showed that the decreased serum fatty acid concentration was the most significant feature in the CENs, supporting our observations from metabolic modeling results. Our study suggested a new signature in exceptional longevity.

## 2 RESULTS

### 2.1 Developing genome‐wide precision metabolic modeling method

**FIGURE 1:**
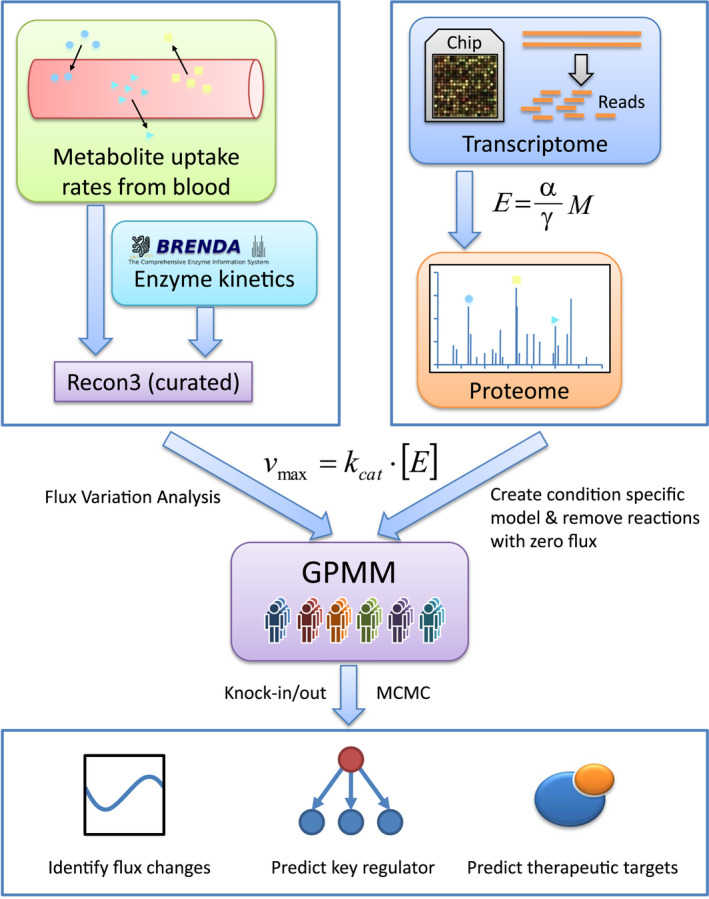
Flowchart of genome‐wide precision metabolic modeling. A generic human metabolism model (Recon 3D) was first curated from the literature, and transcriptome data were then used to estimate enzyme abundance using a steady‐state mathematical model. Next, a reducing model was constructed, and the upper bound of each reaction was calculated using the product of the concentration and turnover number (Kcat) of its enzyme. Finally, flux variability analysis (FVA) was performed to reconstruct individual models, and Markov Chain Monte Carlo (MCMC) sampling was used to detect metabolic differences and key regulators

In the present study, we developed a genome‐wide precision metabolic modeling (GPMM) method to address the “low accuracy” challenge. The method quantitatively integrates the enzyme kinetics information from knowledge bases and the enzyme levels from transcriptome and proteome data into metabolic models (Figure 1 and Figures S1–S3). Specifically, we first curated the generic human metabolic model (Recon 3D; Brunk et al., 2018) (Table S1) and set the upper bounds for the main nutrient uptake rates in blood using information from the literature. To reduce noise from reactions without enzyme kinetics information (the turnover number, Kcats), we then constructed a reduced Recon 3D model to maximize the number of reactions with Kcats and minimize the number of reactions without Kcats (Table S1). We next quantitatively integrated the enzyme kinetic parameters and gene expression levels to constrain the upper and lower bounds of each reaction (Figures S1–S3). According to Michaelis–Menten kinetics, the upper bound of a reaction is the product of the concentration and turnover number (Kcat) of its enzyme. Finally, we used flux variability analysis (FVA) to obtain individual models by removing the reactions with zero flux and then performed Markov Chain Monte Carlo (MCMC) sampling to identify metabolic changes and key regulators.

**FIGURE 2:**
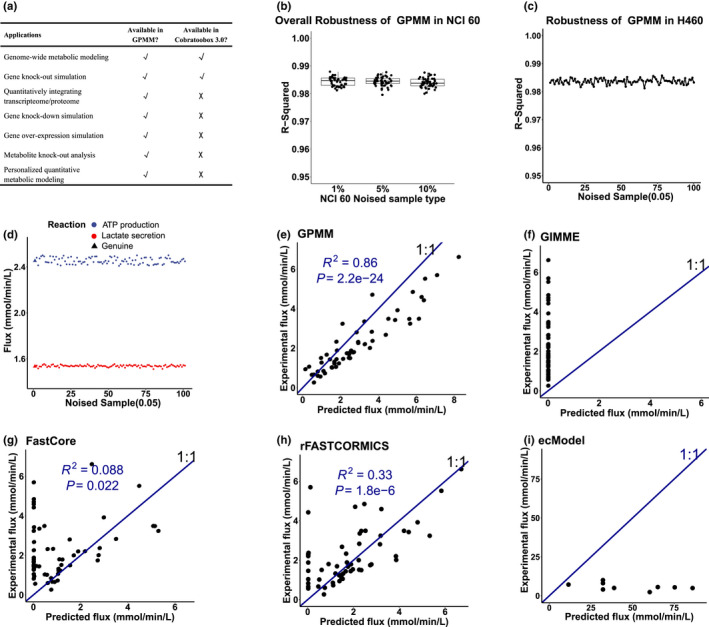
Benchmark analysis of GPMM. (a) Main applications of the GPMM toolbox and comparison with COBRA Toolbox 3.0. (b) Pearson correlation of fluxes between the noise‐induced gene expression and the genuine samples using GPMM in NCI‐60 cell lines. (c) The Pearson correlation between 5% noise‐induced gene expression and genuine samples in the H460 cell line 100 times. (d) Variations in two important fluxes (ATP production and lactate secretion) in cancer cells after inducing 5% gene expression noise using GPMM. (e–i) Comparisons between predicted metabolic fluxes and experimentally measured lactate fluxes in NCI‐60 cells using GPMM (e), GIMME (f), Fastcore (g), rFASTCORMICS (h) and ecModel (i). GPMM, Fastcore, and rFASTCORMICS had *R* ^2^ values of 0.86, 0.088 and 0.33, respectively, whereas GIMME failed to predict lactate secretion. ecModel has the ability to predict lactate secretion, but the magnitude of the predicted fluxes is different from the experimental values. Note: the ecModel reconstruction and the flux detection were derived from Zenodo (https://doi.org/10.5281/zenodo.3577466), and only 11 ecModels are available

Notably, GPMM enabled several in silico analyses that were not included in the state‐of‐art COBRA methods toolbox (COBRA toolbox v3.0), and hence can be broadly applicable in metabolic engineering and therapy (Figure 2a and Table S2). These include not only genome‐wide single and combinatorial knock‐in and knock‐out analysis, but also quantitative inhibition and activation analysis to identify key regulators for target discovery (Figure 2a and Table S2). In addition, GPMM can be applied to conduct personalized metabolic modeling for precision medicine (Figure 2a and Table S2).

### 2.2 GPMM robustly and precisely captured the experimentally measured fluxes

Since the input transcriptome may carry out noise from the experimental or mapping procedures, we analyzed the robustness of GPMM to demonstrate whether GPMM has the ability to tolerate gene expression noise. We first constructed a noise‐induced transcriptome by adding random values (viz. artificial noise) into the original expression data (viz. the genuine expression values) of each gene. Specifically, we induced 1%, 5%, and 10% noise into the gene expression data of NCI‐60 cell lines to construct noise‐induced transcriptomes. Then, we performed the metabolic modeling on the genuine and the noise‐induced expression datasets, and then compared the obtained flux results between both datasets. If a method is robust, this method should be able to tolerate a certain extent of noise on quantified gene expression; then, the correlation (measured by *R* ^2^ or *R*‐squared) between the genuine and noise‐induced samples should approach 1. The results showed that the *R*‐squared in each cell line is larger than 0.98 under either 1%, 5%, or 10% noise (Figure 2b). To further investigate the robustness of GPMM, we next performed multiple random sampling to investigate whether GPMM is still robust at different sampling times (Figure 2c). We induced 5% gene expression noise to the H460 (one of NCI‐60 cell lines) for 100 times and performed metabolic modeling using GPMM. We obtained an average *R* ^2^ of 0.984 (Figure 2c) between the genuine and the noise‐induced samples. Some important fluxes in cancer cells, such as ATP production and lactate secretion, were also consistent among these 100 simulations (Figure 2d). These results indicated that GPMM is a robust method.

To benchmark the performance of GPMM, we chose to utilize the transcriptome and metabolic flux (uptake and secretion) data of the NCI‐60 cell lines (Table S3; Jain et al., 2012). Four other methods commonly used for metabolic modeling, GIMME (Blazier & Papin, 2012), Fastcore (Vlassis et al., 2014), rFASTCORMICS (Pacheco et al., 2019), and ecModel (Robinson et al., 2020; Sánchez et al., 2017), were chosen as comparisons. We applied each of the four methods to the transcriptome data of the NCI‐60 cells and compared the computationally calculated metabolic fluxes with the reported experimental measurements. The results showed that the GPMM method had a much higher correlation between the predicted and experimental values (Figure S4a, *R* ^2^ = 0.72, *p* = 2.3e‐106) than GIMME (Figure S4b, *R* ^2^ = 0.011), Fastcore (Figure S4c, *R* ^2^ = 0.31), rFASTCORMICS (Figure S4d, *R* ^2^ = 0.49), and ecModel (Figure S4e, *R* ^2^ = 0.27).

The Warburg effect, indicated by an increase in lactate secretion, is one of the most important cancer hallmarks in NCI‐60 cells (Jain et al., 2012). We thus compared the predicted lactate secretion fluxes with the experimental values, and found that GPMM well‐predicted the secretion of lactate in NCI‐60 cells (*R* ^2^ = 0.86, *p* = 2.2e‐24) (Figure 2e). For other four methods, neither GIMME nor Fastcore could predict lactate secretion (Figure 2f,g). One of the up‐to‐date methods, rFASTCORMICS, returned reasonable results with the *R* ^2^ of 0.33 (Figure 2h). However, the other up‐to‐date method, ecModel, fails to quantitatively predict lactate secretion (Figure 2i), although its overall prediction performance is reasonable (Figure S4e). These results showed that GPMM can precisely capture the experimentally measured fluxes and significantly outperformed the existing methods.

### 2.3 Metabolic modeling reveals elevated fatty acid oxidation as the most significant metabolic feature in centenarians

To shed light on the metabolic characteristics of the CENs to better understand why these individuals are able to delay or avoid many serious age‐related diseases that afflict the normal population (Evert et al., 2003), we aimed to study the metabolism of longevity in a CEN cohort sampled from Hainan Province, China. The cohort included 76 CENs, 54 centenarian‐children (F1s), and 41 spouses of centenarian‐ children (F1SPs; Table 1), whose RNA‐sequencing data were reported in our previous study (Xiao et al., 2018).

**TABLE 1:** Overall population attributes of the Hainan centenarian cohort

Category	CEN	F1	F1SP	*p*1(CEN vs. F1SP)	*p*2(CEN vs. F1)	*p*3(F1 vs. F1SP)
Sample size	76	54	41	NA	NA	NA
Age	102.2 ± 2.4	63.2 ± 7.7	60.0 ± 6.6	**<0.001**	**<0.001**	**0.04**
Gender: Female (male)	58 (18)	3 (51)	40 (1)	**0.003**	**<0.001**	**<0.001**
Live independence: yes (no)	73 (3)	53 (1)	41 (0)	0.55	0.64	0.99
Diastolic blood pressure (mmHg)	146.0 ± 20.1	138.3 ± 19.2	137.9 ± 18.0	**0.03**	**0.03**	0.92
Systolic blood pressure (mmHg)	83.2 ± 11.8	80.8 ± 21.4	86.1 ± 11.3	0.19	0.46	0.12
Blood glucose (mmol/L)	5.98 ± 1.26	6.43 ± 1.28	6.70 ± 2.96	0.15	0.06	0.6
TC	4.68 ± 0.93	5.02 ± 1.25	5.49 ± 1.60	**0.005**	0.09	0.13
TG	3.73 ± 1.92	3.96 ± 2.13	4.44 ± 2.56	0.14	0.53	0.35
HDL	1.47 ± 0.36	1.51 ± 0.51	1.65 ± 0.27	**0.004**	0.37	0.19
LDL	2.45 ± 0.87	2.76 ± 1.08	3.02 ± 1.43	**0.03**	0.1	0.35

Notes

The *p*‐values of gender and live independence were calculated using Fisher's test. Other *p*‐values were calculated using *t*‐test. Significant *p*‐values are highlighted by bold font. The unit of TC, TG, HDL, and LDL is μmol/L.

Abbreviations: HDL, High‐density lipoprotein cholesterol; LDL, Low‐density lipoprotein cholesterol; TC, Total cholesterol; TG, Total triglyceride.

**FIGURE 3:**
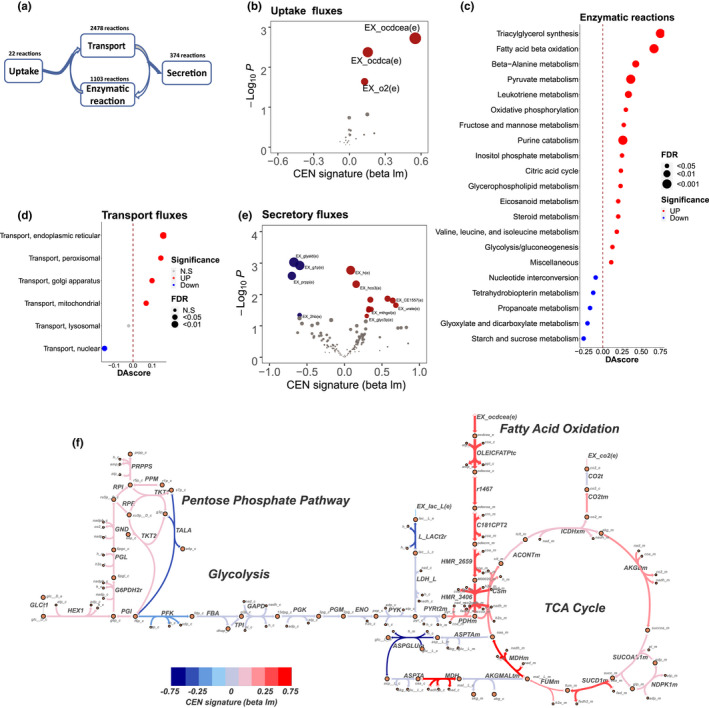
Genome‐wide metabolic modeling of white blood cells from centenarians (CENs) using GPMM. (a) Schematic of four functional components of metabolic modeling. (b) Volcano plot of uptake reactions. The *X*‐axis and *Y*‐axis are beta and *p*‐values of CEN signatures using a linear model. (c) Differential abundance (DA) score plot of significantly changed enzymatic reaction component. (d) DA score plot of transport components. *Note*: transport in endoplasmic reticular was the most significant subsystem in the CENs. (e) Volcano plot of secretion reactions. (f) Metabolic map of core carbon metabolic fluxes. Red and blue represent up‐ and down‐regulated metabolic fluxes in the CENs, respectively

We next applied GPMM to study the metabolic features of longevity in this cohort. In total, we developed 171 individual GPMM metabolic models based on white blood cell transcriptome information. Each model contained 3977 reactions, which could be classified into four functional components (Brunk et al., 2018): nutrient uptake (22 reactions), metabolite transport (2478 reactions), enzyme‐catalyzed reaction (1103 reactions), and secretion and demand reaction (374 reactions) (Figure 3a and Tables S4–S6). By comparing the differences in metabolic characteristics between the CENs and younger controls (viz. F1SPs) and adjusted for age and gender effect, we obtained 343 upregulated and 90 downregulated fluxes. We observed that the overall CEN flux signature was slightly negatively correlated with the aging effect (*r* = −0.15, *p* = 7.1e‐12) (Figure S5a,b), suggesting that the CENs contain some signatures that are different from the ones associated with age. The most striking signature in all four metabolic processes consistently indicated that long‐chain fatty acid beta‐oxidation (FAO) was elevated in the CENs. For illustration, in the nutrient uptake component, long‐chain fatty acids (viz. octadecanoate and octadecenoate) and oxygen uptake were significantly elevated in the CENs (Figure 3b); in the metabolite transport component, transport in the subcellular organelles for long‐chain fatty acid oxidation (viz. peroxisome and mitochondria) were also elevated in those long‐lived individuals (Figure 3d). Similarly, the enzymatic catalyzing component showed that cellular fatty acid storage (viz. triacylglycerol synthesis), FAO, pyruvate metabolism, branch amino acid metabolism, tricarboxylic acid (TCA) cycle, and oxidative phosphorylation were all elevated in the CENs (Figure 3c). Consistently, in the secretory and demand components, we found that the CENs released more carbon dioxide and fewer TCA intermediate metabolites (Figure 3e).

Given the crucial role of FAO in carbon catabolism (Brunk et al., 2018), we explored the upstream and downstream reactions of this process to determine whether the observed elevation was restricted to FAO or existed in other carbon catabolism pathways. Surprisingly, we found that the upstream reactions of FAO, including fatty acid uptake, activation, and transport, were all elevated in the CENs (Figure 3f). Downstream reactions of FAO, half of the tricarboxylic acid cycle (TCA cycle) fluxes (4 out of 8 reactions) and over half of the oxidative phosphorylation complexes (3 out of 5 reactions) were significantly increased in the CENs (Figure 3f and Figure S6a). Consistent with these observations, total cellular ATP production capacity was also significantly enhanced (*p* = 0.032) in the CENs (Figure S6b).

### 2.4 Serum metabolomics supports the metabolic modeling observations

**FIGURE 4:**
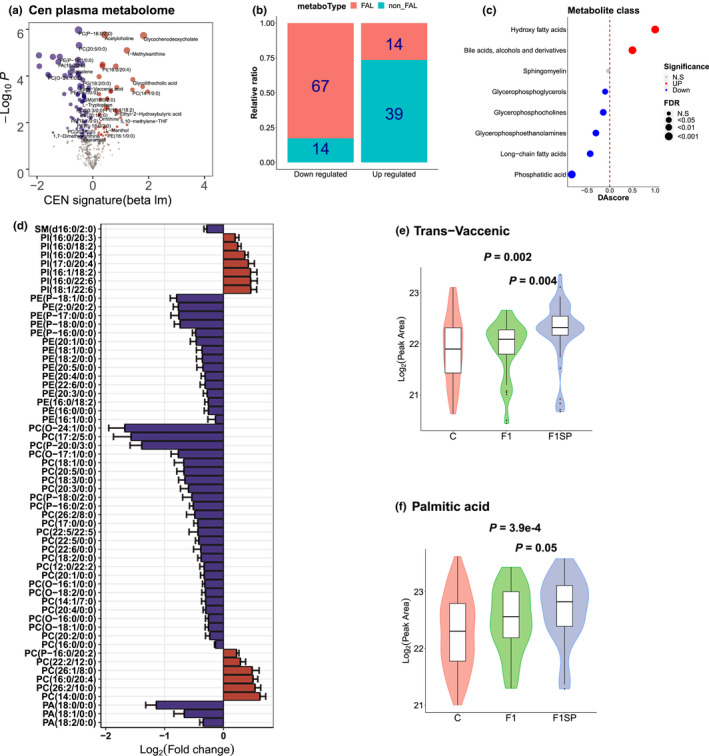
Metabolism profile in the CEN serum. (a) Volcano plot of changes in plasma metabolites (*N* = 505) in the CENs. (b) Relative ratio of fatty acid‐like (FAL) upregulated and downregulated metabolites in CENs. (c) Represents the metabolite class enrichment analysis using the DAscore method. (d) Abundance profile of significantly changed fatty acid‐like (FAL) metabolites, including phosphatidic acids (PAs), phosphatidylethanolamines (PEs), phosphatidylcholines (PCs), phosphatidylinositol (PIs), and long‐chain fatty acid sphingomyelin (SM) in the CENs. (e and f) Abundance of trans‐vaccenic and palmitic acids among the CENs, centenarian offspring (F1), and spouses of centenarians’ offspring (F1SPs)

Because a higher systemic FAO leads to higher uptake and consumption of fatty acids in tissues (Jang et al., 2016), we hypothesized that the serum long‐chain fatty acid concentration should be decreased in the CENs. By generating and analyzing the serum metabolomics data of the same longevity cohort, we obtained 505 metabolites. After removing the metabolites associated either with aging or with gender effect, we identified 83 downregulated and 53 upregulated metabolites in the CENs (Figure 4a). Among the downregulated metabolites, 80.7% (67/83) were fatty acid‐like (FAL) metabolites (Figure 4b). This value remained stable after upregulated FAL metabolites were considered as well (82.7%, 67/81) (Figure 4b). Interestingly, differential abundance score (DA) analysis (Hakimi et al., 2016) showed that 71% (5/7) of FAL families were significantly downregulated in the CENs, including phosphatidic acids (PAs), phosphatidylethanolamines (PEs), phosphatidylcholines (PCs), and long‐chain fatty acid sphingomyelin (SM) (Figure 4c). Specifically, 100% (3/3) of PAs, 100% (15/15) of PEs, 81.2% (26/32) of PCs, and 100% (1/1) of SMs were significantly decreased in the CENs (Figure 4d). In addition to FAL metabolites, free long‐chain fatty acids (e.g., trans‐vaccenic and palmitic acids) were also significantly decreased in the CENs (*p* = 0.002 and 3.9e‐4, respectively) (Figure 4e,f). Notably, we also observed that F1s had significantly lower trans‐vaccenic levels (Figure 4e, *p* = 0.004) and significantly lower palmitic acid levels (Figure 4f, *p* = 0.05) than F1SP. These results suggested that several fatty acid features from CENs, such as decreased free fatty acids, are likely heritable. Intriguingly, among the upregulated metabolites, the most significant ones were bile acids, a group of metabolites for fatty acid absorption (Figure 4c). These results suggest that the decreased serum fatty acid concentration was the most significant feature in our centenarian metabolomics data. This observation also explains our previous epidemiological survey, which found that total cholesterol is decreased in the CENs compared with F1SPs (He et al., 2014). A similar result was also obtained by analyzing the clinical data of the same longevity cohort studied here (Figure S7).

## 3 DISCUSSION

Identifying metabolic signatures in centenarians is important for healthy aging. Constraint‐based reconstruction and analysis (COBRA) is a promising method that can capture metabolic signatures in health and diseases, but has been a long‐standing challenge to quantitatively predict molecular phenotypes (Lewis et al., 2010; O’Brien et al., 2015). In this study, we present a solution to this critical problem by incorporating quantitative restraints on each reaction in genome‐scale modeling. Here, the maximum rate of each reaction is set at Kcat*[*E*], where Kcat is mined from enzyme databases and the concentration of the enzyme is either measured by proteomics or estimated from transcriptomics (under the steady‐state approximation). In a large benchmark study, this method (GPMM) successfully characterized metabolic reprogramming in NCI‐60 cancer cell lines (Jain et al., 2012); it dramatically improved the performance of the modeling with an *R* ^2^ of 0.86 between the predicted and experimental measurements over the performance of existing methods (Figure 2 and Figure S4). As most parameters and datasets are precalculated, GPMM is easy to use, and the only required input is the transcriptome (RNA‐sequencing or microarray). Therefore, GPMM is able to systematically analyze cellular metabolic profiles using only the transcriptome and enables broad computational studies on discovering disease mechanisms.

Previous studies indicated that the existing continued methods, such as PRIME (Yizhak et al., 2014) and RegrEX (Robaina Estévez & Nikoloski, 2015), are less robust than the methods that utilize discretization workflows, such as rFASTCORMICS (Pacheco et al., 2016, 2019). However, the results showed that our developed quantitative method can robustly and well predict experimentally measured fluxes. The reason may be because we not only translated the transcripts to proteome data using a simple but efficient model, but also used enzymatic parameters to restrain the maximum rate for each reaction. In addition, to avoid the effect of the unconstrained reactions on the metabolic simulation, we also reduced the generic models (i.e., Recon3D) to maximize the number of reactions with Kcats and minimize the number of reactions without Kcats. Similar to rFASTCORMICS, GPMM can also use the secretion information to improve the model performance by adding the secretion reaction information in the exchange input file. These improvements thus largely overcome the performance and robustness issues of the existing methods. Therefore, the dramatic improvement of GPMM will enable many computational studies to discover biomedical mechanisms.

Utilizing this method, we studied the metabolic profiles of CENs and identified the elevated fatty acid oxidation as the most significant metabolic feature in the CENs. As the input of GPMM is the transcriptome, we investigated the main beta‐oxidation genes from Recon 3D (Brunk et al., 2018) and found that 11 of 14 (78.6%) FAO‐related genes were slightly upregulated in the CENs (Figure S8a), including four essential peroxisomal beta‐oxidation genes (*EHHADH*, *HSD17B4*, *ACAA1*, *and ACOX1*) and five key mitochondrial beta‐oxidation genes (*HADHB*, *ACAA2*, *ECHS1*, *ACADL*, *and ACADVL*) (Figure S8a). We also observed that 9 of 14 (64.3%) FAO‐related genes were slightly downregulated with aging in F1SP samples (Figure S8b). These results support our findings that the elevated fatty acid oxidation as a metabolic signature in the CENs.

Furthermore, we also obtained additional evidence on the CENs’ serum metabolomic data that most FAL metabolites, including phosphatidic acids (PAs), phosphatidylethanolamines (PEs), phosphatidylcholines (PCs), and long‐chain fatty acid sphingomyelin (SM), were significantly downregulated in the CENs (Figure 4d). Consistent with previous metabolomic studies that a larger percentage of detected FAL metabolites (17/22, 77.2%) was decreased in the CENs (Collino et al., 2013), we also found that most of the FAL metabolites (67/83, 80.7%) were decreased in our CEN cohort. Similarly, Pradas et al. (2019) reported that the PEs displayed reduced levels in CENs, a result also replicated in our study. Interestingly, contrast to the previous observations that l‐carnitine, an essential transport of long‐chain fatty acids from the cytosol to the mitochondrial matrix, exhibited a significantly decreased level with aging (Calabrese et al., 2010; Noland et al., 2009), we also investigated but found that the level of l‐carnitine was not decreased in CENs, suggesting that the aging‐related decrease in FAO did not occur in the CENs. Taken together, these findings support that an increased FAO activity exists in the CENs.

Previous studies have shown that the offspring of centenarians inherit part of survival advantages from their long‐lived parents, and are also used to explore the health‐protective mechanism of human aging (Brooks‐Wilson, 2013; Xiao et al., 2018). Therefore, we compared the estimated metabolic signatures between F1s and F1SPs. The result showed that the overall CEN‐specific flux signatures were significantly positively correlated with the Flux signatures in F1s (*r* = 0.45, *p* = 1.8e‐185; Figure S9a). Specifically, most of significantly upregulated FAO‐related reactions observed in the CENs, including fatty acid beta‐oxidation, perisomal transport, citric acid oxidation, oxidative phosphorylation, and ATP production, are also upregulated in F1s (20 of 27, 74%, Figure S9b). Consistently, serum metabolism results also showed that most of down‐regulated metabolites in serum are FALs (12 of 20, 60%); and most of FALs with significant differences between the two groups are downregulated (12 of 19, 63%) in F1s (Figure S9c). Interestingly, we also observed that F1s has lower free fatty acid levels (i.e., trans‐vaccenic and trans‐vaccenic levels) than F1SP (Figure 4e,f). Given that F1s might have a higher probability of long lifespan than F1SPs, these results added further support to our conclusion that the elevated fatty acid oxidation is a signature involved to healthy human aging and longevity.

There are many studies in both model organisms and humans showing associations between FAO decline and aging (Gong et al., 2017; Levadoux et al., 2001; Short et al., 2005). However, whether FAO also declines in the CENs, the paradigm of healthy human aging, remains unclear. Interestingly, multiple lines of evidence from our study argues for an enhanced FAO in the CENs, which well explains the previous observations that decreased fatty acid levels, especially the PEs, were found in centenarians’ serum (Pradas et al., 2019). In addition, impaired FAO is frequently observed in many age‐related diseases, including atherosclerosis (Freigang et al., 2013) and diabetes (Wei et al., 2016). Importantly, elevated FAO is reported to be causally associated with metformin‐induced longevity in *Caenorhabditis elegans* (Pryor et al., 2019). Elevated fatty acid beta‐oxidation related genes extend the lifespan of worms (Lee et al., 2012). Collectively, these results suggest that the elevated long‐chain FAO function in the CENs, at least in female CENs, represents a “healthy” metabolic profile of longevity, which may convey survival advantages to long‐lived individuals by reducing lipid accumulation and lowering the risks of common age‐related diseases, especially those involved in lipid metabolic disorders.

In summary, we have developed a novel systems biology approach to effectively integrate omics data in the modeling of metabolic mechanisms in human health and disease. This approach dramatically improved the performance over the existing methods. Our method thus immediately enables many computational studies on discovering disease mechanisms and candidate drug targets, as well as further developments of the algorithms. We applied this method to investigate the metabolic profiles of CENs, and suggested the enhanced fatty acid oxidation as a novel metabolic signature of healthy aging in exceptional longevity.

Nevertheless, there are some limitations that need to be overcome in the future. (i) In GPMM method, the reduced model by maximizing the number of reactions with known Kcats could introduce potential biases, as less well studied enzyme‐related reactions and pathways are less likely to be included in the reduced model. (ii) Although the GPMM model displays much better performance in predicting metabolic flux, its ability in dealing with the low flux levels (i.e., <0.1 mmol/min/L) is still limited. Fixing this limitation will be the major objective of the next version of GPMM, which would definitely be of help in improving the performance of the method in metabolic modeling. (iii) Although we used a series of analyses, including two linear models, to evaluate the potential effect of age and remove any signals associated with age, it is still possible that some age effect persists in our results, simply owing to the fact that some age signal within the F1SP population might not be associated with age in the CEN group. (iv) Although our results suggest that the CENs likely display increased lipid metabolism, which gets further support from serum metabolome, whether this signature can represent the whole body of these long‐lived individuals awaits further investigation, largely due to the fact that our transcriptome data are obtained from the peripheral white blood cells.

## 4 MATERIALS AND METHODS

### 4.1 Genome‐wide precision modeling of the metabolism

#### 4.1.1 The flowchart of GPMM

The GPMM method was designed to integrate enzyme kinetics into constraint‐based genome‐wide metabolic modeling. It integrated the knowledge‐based human metabolic reconstruction model (Recon 3D) (Brunk et al., 2018) with enzyme kinetics, transcriptomics, proteomics, and metabolomics data to perform metabolic modeling.

The GPMM flowchart is shown in Figure 1. The generic human metabolism model (Recon 3D) was first curated from published literature, with the uptake upper bounds of exchange reactions in blood obtained from the literature. Second, transcriptome data were used to estimate enzyme abundance with a mathematical model. Third, the Gene Inactivity Moderated by Metabolism and Expression (GIMME) method (Blazier & Papin, 2012) was used to reduce Recon 3D to the maximal usage of the quantitative upper bounds of the reactions. Fourth, FVA was performed to remove reactions with zero flux and reconstruct the tissue‐specific GPMM. Finally, the reconstructed model was simulated using in silico knock‐in and knock‐out and MCMC sampling methods to detect metabolic differences and key regulators.

#### 4.1.2 Knowledgebase curations

To reconstruct genome‐wide metabolic models (GEMs) by the GPMM approach, we collected several relevant knowledge bases. The knowledge‐based human metabolic model was obtained from Recon 3D (Brunk et al., 2018). The enzyme Kcat values were downloaded from BRENDA (Placzek et al., 2017). Serum metabolite concentrations were obtained from the Human Metabolome Database (HMDB) (Wishart et al., 2018).

Next, we manually curated the global human metabolic network of Recon 3D using thermodynamic analysis (Martinez et al., 2014) and the precured Recon 2 model (Quek et al., 2014). Because adenosine monophosphate (AMP) cannot be directly changed into adenosine triphosphate (ATP) in any reaction, some reversible reactions, such as FACOAL150 and RE1514M, were curated as irreversible. The curated Recon 3D model is shown in Table S1.

#### 4.1.3 Setting quantitative upper and lower bounds of biochemical reactions

For each biochemical reaction, the flux of any reaction has the following equation:

(1) $$V\leq V_{\max}=\left[E\right]\times \mathrm{Kcat}$$

where *V* is the flux of a reaction, *V*_max_ is the maximum reaction rate according to Michaelis–Menten kinetics, [*E*] is the enzyme concentration, and Kcat is the turnover number of the enzyme. The Kcat values of human enzymes were obtained from the BRENDA database (Placzek et al., 2017). If an enzyme had multiple Kcat records, their median was used. Where experimental data were missing, we used Kcats from other species.

We obtained 2602 Kcat records in the 4352 reactions with an EC number in Recon 3D (Table S1). Although 42% reactions with an EC number lacked Kcat records, the enzyme abundance percentage was smaller than 10% (Figure S1).

A previously published method, named GIMME (Blazier & Papin, 2012), was used to reduce the Recon 3D model to maximize the number of reactions with Kcats and minimize the number of reactions without Kcats. The GIMME objective functions were set to ATP production and biomass reaction, as described in previous studies (Blazier & Papin, 2012; Nam et al., 2014). Finally, we obtained a reduced Recon 3D model, with 5134 metabolites, 7871 reactions (including 3750 transport, 1787 exchange/demand, and 3168 enzyme‐related reactions), and 2248 genes. This retained 88% (5134/5835) metabolites and 74% reactions (7871/10,608) in the original model (Recon 3D). Only 566 of the 3168 (17.8%) enzyme‐related reactions in reduced model lacked Kcat records.

#### 4.1.4 Predicting enzyme abundance using gene expression data

Recently, a simple but efficient mathematical model was proposed to predict protein abundance using gene expression data (Wilhelm et al., 2014). Changes in enzyme abundance can be determined by the number of proteins synthesized from mRNA minus the number of proteins degraded. In the steady‐state, we have following equation:

(2) $$\frac{dE}{dt}=\alpha M‐\gamma E$$

where *E* and *M* are the enzyme and corresponding mRNA abundances, respectively; *α* is the enzyme synthesis rate from mRNA; and *γ* is the enzyme degradation rate.

Thus, in the steady‐state, we can predict enzyme abundance as follows:

(3) $$E=\frac{\alpha }{\gamma }M$$

To estimate the α/γ ratio, we downloaded microarray data of 12 normal tissues with GSE7307 (http://www.ncbi.nlm.nih.gov/geo/query/acc.cgi?acc=GSE7307) and RNA‐seq data of 15 normal tissues from the Human Protein Atlas Dataset (Uhlen et al., 2015). We also obtained the corresponding protein abundance data from the MOPED database (Montague et al., 2014) with the unit of nmol/L. MOPED uses the human body map dataset and estimates protein concentration from protein abundance (Montague et al., 2014). Thus, the α/γ ratio was estimated using the median ratio of protein/mRNA across multiple tissues.

The correlation between the transcriptome and proteome is usually quite low (Pearson correlation of 0.4–0.5, Figure S2). Notably, using the steady‐state kinetic method, the Pearson correlation between the predicted proteome and experimental measurements reached 0.8–0.9 (Figure S3), indicating that protein abundance could be correctly estimated using transcriptome data.

#### 4.1.5 Steps of the metabolic modeling

First, enzyme abundance was predicted using the above mentioned mathematical model. Second, the upper bound activity of enzyme‐related reactions was calculated by multiplying the Kcat value by the enzyme concentration. For the gene‐protein‐reaction (GPR) relationships in Recon 3D, some reactions have more than one enzyme. Thus, we calculated the upper bound activities of these reactions as follows: (a) summed enzyme activity when the GPR had a Boolean logic of “OR”, and (b) minimized enzyme activity when the GPR had a Boolean logic of “AND.” Oxidative phosphorylation is essential for ATP production, and all eight essential oxidative phosphorylation reactions (including ATPS4mi, CYOOm2i, CYOR_u10mi, NADH2_u10mi, r0205, CYOOm3i, FADH2ETC, GLYC3PFADm) require more than 20 enzymes to catalyze (Brunk et al., 2018). Any missing data will result in inactivity of these reactions. Thus, we did not apply the quantitative upper bounds to these eight essential oxidative phosphorylation reactions, and instead set them to unlimited.

Second, after setting the quantitative upper bounds of the reactions and the uptake values, we performed flux variation analysis (FVA) to obtain the maximum and minimum fluxes for each reaction. Some reactions have both the maximum and minimum fluxes of zero, thus cannot carry a flux. The individual models were then reconstructed by removing the reactions that could not carry flux in the FVA. Large‐scale FVA was performed using our recently published efficient constraint‐based metabolic modeling toolbox FastMM (Li et al., 2020) (https://github.com/GonghuaLi/FastMM).

Third, hit‐and‐run Monte Carlo simulation was performed to obtain the distribution of each flux in each model via the “ACHRSampler” function in the Cobra toolbox 3.0 (Heirendt et al., 2019). To compare flux in different metabolic models, we averaged and summarized the MCMC sampling fluxes using the “summarize_PQMM_result” function in our developed GPMM toolbox. We finally obtained an average flux matrix, where row and column names represent reaction names and sample identities, respectively.

The GPMM toolbox is available at https://github.com/GonghuaLi/GPMM.

### 4.2 Benchmark study of GPMM

#### 4.2.1 Collection of experimental fluxes

The experimental flux dataset of NCI‐60 cells was derived from Jain et al. (2012). The dataset contained 59 cell line flux data, with each cell line containing 119 Recon 3D exchange reaction fluxes (Table S3), where the unit of fmol/cell/h was converted to mmol/L/min. To obtain fluxes with high experimental confidence, we removed the outliers and those reactions with the median fluxes among different cell lines <1e‐3 mmol/L/min and finally retained 1128 uptake and 381 secretion fluxes from this database.

#### 4.2.2 Predicting fluxes of NCI‐60 cells using GPMM

The gene expression dataset (RNA‐seq) was downloaded from the CellMiner website (https://discover.nci.nih.gov/cellminer/loadDownload.do), and the uptake rate for each cell was obtained from the above experimental fluxes (Jain et al., 2012). Using the GPMM toolbox mentioned above, and setting ATP production and biomass reaction as the optimized functions, genome‐wide precision metabolic modeling of NCI‐60 cells was performed to obtain the flux matrix. The predicted secretion fluxes were then compared with the experimental dataset to evaluate the overall performance of the GPMM.

#### 4.2.3 Predicting fluxes of NCI‐60 cells using GIMME

The quantitative gene expression of NCI‐60 was the first transformed into qualitative present/absent logical values using a Fragments Per Kilobase of transcript per Million mapped reads (FPKM) cutoff = 3.0. Metabolic models were then reconstructed using the “GIMME” function in the Cobra toolbox 3.0 (Heirendt et al., 2019). Next, MCMC sampling was conducted to obtain the distribution of fluxes, and the average flux for each reaction was then calculated to obtain the GIMME‐based flux matrix.

#### 4.2.4 Predicting fluxes of NCI‐60 cells using Fastcore

The consistent Recon 3D model was first constructed using the “fastcc” function in the Fastcore toolbox (Vlassis et al., 2014) with an epsilon of 1e‐4 using the linear solver of cplex (https://www.ibm.com/analytics/cplex‐optimizer). The metabolic models were then reconstructed using the “fastcore” function in the Fastcore toolbox, and MCMC sampling was conducted using the “ACHRSampler” function in the Cobra toolbox 3.0 (Heirendt et al., 2019).

#### 4.2.5 Predicting fluxes of NCI‐60 cells using rFASTCORMICS

rFASTCORMICS is an updated version of Fastcore that uses discretization workflows instead of the heuristic thresholds method (Pacheco et al., 2019). The consistent Recon 3D model was also first constructed using the “fastcc” function in the Fastcore toolbox (Vlassis et al., 2014) with an epsilon of 1e‐4 using the linear solver of cplex (https://www.ibm.com/analytics/cplex‐optimizer). The metabolic models were then reconstructed using rFASTCORMICS (Pacheco et al., 2019), and MCMC sampling was conducted using the “ACHRSampler” function in the Cobra toolbox 3.0 (Heirendt et al., 2019).

#### 4.2.6 Predicting fluxes of NCI‐60 cells using ecModel

The model reconstruction and flux detection of 11 NCI‐60 cell lines were derived from Zenodo (https://doi.org/10.5281/zenodo.3577466). Note: as each constructed ecModel has over 20,000 reactions and has a different model framework from the COBRA toolbox, performing MCMC sampling is difficult. Therefore, the fluxes for each ecModel were estimated using the suggested method “minProSimulation” from Human 1 (Robinson et al., 2020).

#### 4.2.7 Comparisons of the predicted and experimentally measured fluxes

The predicted secretion fluxes using different methods, that is, GPMM, GIMME, and Fastcore, were compared with the experimental flux datasets as mentioned above. To avoid linear optimization precision error, we removed the fluxes with absolute values smaller than 1e‐6 mmol/L/min. Pearson correlations between predicted fluxes and experimental measurements were calculated to compare the performance of the different methods.

#### 4.2.8 Robustness analysis of GPMM

We first constructed noise‐induced transcriptomes by adding random numbers (viz. artificial noise) to the original expression data (or the genuine transcriptome) of each gene. Specifically, 1%, 5%, and 10% noise was induced in each NCI‐60 cell line transcriptome. In these processes, the noise inducing is produced from a uniform distribution of [0.99, 1.01] for 1% noise, [0.95, 1.05] for 5% noise and [0.90, 1.10] for 10% noise. For example, in the 5% noise translation procedure, if a gene in a cell line has a gene expression (e.g., fpkm) of 1.0, the adding random number of this gene is ranged from −0.05 to 0.05, such as 0.03; then, the noised‐induced gene expression is a number ranged from 0.95 to 1.05, such as 1.03. Second, we performed the metabolic modeling on the genuine and the noise‐induced transcriptomes and compared the obtained flux results to evaluate GPMM robustness. In addition, to further test whether multiple sampling affects robustness, we also induced 5% noise to the gene expression of H460 cell line 100 times and performed metabolic modeling to determine the stability of GPMM.

### 4.3 The Chinese centenarians study

A total of 171 individuals from longevity families, consisting of 76 centenarians (CENs), 54 centenarian‐children (F1), and 41 spouses of centenarian children (F1SP), were recruited from Hainan Province, China, as part of the study of centenarians in southern China (He et al., 2014). The research protocol was approved by the Ethics Committee at Kunming Institute of Zoology, Chinese Academy of Sciences. Written informed consent was obtained from each of the participants prior to the study.

As shown in Table 1 and Table S4, 96% of CENs lived independently (e.g., eating, walking, and talking). Compared with F1SPs, CENs had significantly higher diastolic blood pressure (146.0 vs. 137.9, *p* = 0.03), similar systolic blood pressure (83.2 vs. 86.1 *p* = 0.19), slightly lower blood glucose (5.98 vs. 6.70, *p* = 0.14, *t*‐test), lower total cholesterol (4.68 vs. 5.43 *p* = 0.009, *t*‐test), and lower low‐density lipoprotein cholesterol (2.45 vs. 2.97, *p* = 0.043, *t*‐test). These results are also consistent with our previous studies, where levels of risk factors for cardiovascular diseases, including blood glucose, triglyceride, and total cholesterol, were significantly lower in the CENs than those of the general older population from the same province, and the diagnoses of type 2 diabetes mellitus, hypertriglyceridemia, and hypertension were lower in the CENs than Chinese national levels (He et al., 2014). The relatively healthy status of CENs suggests that they can serve as a good model for healthy aging studies.

For transcriptome analysis, peripheral blood samples were treated with red blood cell lysis buffer (Tiangen Biotech) and then centrifuged at 1800 *g* for 10 min to isolate white blood cells. For metabolomics and proteomics measurements, peripheral blood samples were allowed to clot at room temperature for 30 min and then centrifuged for 10 min at 1500 *g* to extract the serum.

### 4.4 Genome‐wide precision modeling of the metabolism of centenarians

#### 4.4.1 Metabolic modeling

Gene expression (FPKM) levels in 170 individuals from the Hainan longevity cohort, including 76 CENs, 52 centenarian‐children, and 42 spouses of centenarian‐children (F1SPs), were derived from our previously published data (Xiao et al., 2018). The upper bounds of the white blood cell metabolite uptake rates were separated into three categories: nutrient uptake for energy production, cofactors, and iron/oxygen uptake (Table S5). The nutrient uptake rates, including those of glucose, l‐glutamine, and fatty acids, were derived from published literature (Table S5). The essential amino acid uptake rates were set to a small number, whereas those of cofactors, iron, oxygen, and primers for glycogen synthesis were set to unlimited (Table S5). After preparing the gene expression and nutrient uptake rates, genome‐wide precision metabolic modeling was conducted using our developed GPMM toolbox.

#### 4.4.2 Identification of metabolic flux profiles of centenarians

As the CENs (aged 98–108) were older than the controls (45–75), we used two linear models to distinguish centenarian signatures and aging effects. Model l was applied to CEN and F1SP samples to determine unfiltered centenarian signature:

(4) $$\text{Model 1}:\;\text{flux}∼\text{lm}\left(\text{centenarians}+\text{sex}\right)$$

Model 2 was applied to F1SP samples to determine aging effects:

(5) $$\text{Model 2}:\;\text{flux}∼\text{lm}\left(\text{age}+\text{sex}\right)$$

After the unfiltered centenarian signatures and aging effect were determined, we obtained the actual centenarian signatures by excluding the overlapping fluxes between the unfiltered centenarian signature and the age effect. Therefore, upregulated fluxes in CENs were defined as fluxes with *p* < 0.05 and beta >0 in model 1 but not significant or beta <0 in model 2. Downregulated fluxes were defined vice versa.

#### 4.4.3 Identifying significant metabolic subsystems using differential abundance scores

The differential abundance (DA) score was calculated using previously published methods (Hakimi et al., 2016). For each metabolic subsystem, the *i*th DA score (${\mathrm{DA}}_{i}$) was calculated as follows:

(6) $${\mathrm{DA}}_{i}=\frac{\#\;\mathrm{Up}\;\mathrm{regulated}\;\mathrm{fluxes}‐\#\;\mathrm{down}\;\mathrm{regulated}\;\mathrm{fluxes}}{\mathrm{Total}\;\mathrm{reactions}\;\mathrm{in}\;i\mathrm{th}\;\mathrm{subsystem}}$$

To obtain the significance of DA scores, we used a “bootstrap without replacement” method to calculate *p*‐values. Briefly, we first randomly shuffled the sample labels 1000 times. Second, for each randomly shuffled label, the corresponding random DA scores were calculated using the above formula. We thus obtained 1000 random DA scores for each subsystem. Third, the *p*‐value was calculated as follows:

(7) $$p\left({\mathrm{DA}}_{i}\right)=\left\{\begin{array}{c} sum\left({\mathrm{RandomDAs}}_{i}\;>{\mathrm{DA}}_{i}\right)/{\#\;\mathrm{of}\;\mathrm{RandomDAs}}_{i}\;\;\;\;if\;DA>0\\ sum\left({\mathrm{RandomDAs}}_{i}<{\mathrm{DA}}_{i}\right)/{\#\;\mathrm{of}\;\mathrm{RandomDAs}}_{i}\;\;\;\;\;\;if\;DA<0 \end{array}\right.$$

where ${\mathrm{DA}}_{i}$ is the centenarian DAscore of the *i*th subsystem and ${\mathrm{RandomDAs}}_{i}$ is the random DAscore of the *i*th subsystem. The adjusted *p*‐value was calculated using the false discovery rate (FDR).

### 4.5 Metabolomic analysis of the centenarians

#### 4.5.1 Sample preparation

The collected serum samples were thawed on ice. Samples (100 μl) were extracted with 750 μl of methanol/acetonitrile/water solution (*V*_methanol_:*V*_acetonitrile_:*V*_water_ = 2:2:1), with 30 μl of 1 mg/ml l‐2‐chlorophenylalanineas then added as an internal standard, followed by vortexing for 10 s and sonicating for 10 min on ice. After that, the extract was incubated for 1 h at −20°C. Following centrifugation at 16,000 *g* for 15 min, 350 μl of supernatant was transferred into LC–MS vials and dried with a vacuum concentrator. Finally, the vials were resuspended in 100 μl of methanol/acetonitrile/water solution, and the above process was repeated, with 60 μl of supernatant transferred into a new LC/MS vial. To assess the analytical method, 10 μl of each sample was pooled as a quality control (QC) sample, which was tested during analysis.

#### 4.5.2 LC‐MS/MS

LC‐MS/MS analyses were performed with an ultra‐high‐performance liquid chromatography (UHPLC) system (1290, Agilent Technologies) equipped with a Triple‐TOF 6600 mass spectrometer (MS) (Q‐TOF, AB Sciex). Chromatographic separation was carried out on an ACQUITY UPLC BEH Amide column (1.7 μm, 2.1 × 100 mm, Waters). The mobile phase consisted of 25 mM NH_4_OAc and 25 mM NH_4_OH in water (pH = 9.75) (A) and acetonitrile (B). The optimized UPLC elution conditions were as follows: 0–2.0 min, 85.0%–75.0% B; 2.0–9.0 min, 75.0%–0% B; 9.0–14.0 min, 0% B; 14.0–15.0 min, 0–85.0% B; and 15.0–20.0 min, 85% B. The flow rate was 0.3 ml/min. Sample solution (2 μl) was injected for each run. Mass spectrometry was performed on a Triple‐TOF MS in positive and negative mode and operated in information‐dependent basis (IDA) mode. In each cycle, six precursor ions (intensity >100) were chosen for fragmentation with 35 V collision energy (CE) (15 MS/MS events with a product ion accumulation time of 50 ms each). Ion source gas 1 was 60, ion source gas 2 was 60, curtain gas was set to 30 L/h, source temperature was set to 550°C, and ion spray voltage floating (ISVF) was set to 5500 V or −4500 V in positive or negative modes, respectively.

#### 4.5.3 Data preprocessing and annotation

UPLC‐MS raw data (.wiff) were converted to mzXML, with ProteoWizard Peak exaction, identification, integration, alignment, and retention time correction processed with XCMS (R package, v3.2). The preprocessing results generated a data matrix that consisted of retention time (RT), mass‐to‐charge ratio (*m*/*z*) values, and peak intensity. The R package CAMERA was used for peak annotation after XCMS data processing. The in‐house MS2 database was applied for metabolite identification, and only the metabolites with MS2 >0.8 remained.

#### 4.5.4 Identification of metabolomic profiles of centenarians

Using equations to those shown in flux analysis, we also used two linear models to distinguish centenarian signatures and aging effects in the metabolomic data. Model l was applied to CEN and F1SP samples to determine unfiltered centenarian signatures:

(8) $$\text{Model 1}:\;\text{metabolic abundance}∼\text{lm}\left(\text{centenarians}+\text{sex}\right)$$

Model 2 was applied to F1SP samples to determine aging effects:

(9) $$\text{Model 2}:\;\text{metabolic abundance}∼\text{lm}\left(\text{age}+\text{sex}\right)$$

Then, the actual centenarian signature was calculated by excluding the overlapping metabolites between the unfiltered centenarian signature and the age effect. Therefore, upregulated metabolites in CENs were defined as the log2 transformed metabolic abundance with *p* < 0.05 and beta >0 in model 1 but not significant or beta <0 in model 2. Downregulated metabolites were defined vice versa.

#### 4.5.5 Identifying significant metabolic classes in metabolomic data

Similar to the flux subsystem analysis in Equations (6) and (7). The significance of the metabolic class was also analyzed using the differential abundance (DA) score (for Figure 4c). For each metabolic class, the DA score was calculated as the number of upregulated metabolites minus the downregulated metabolites and then divided by the total number of metabolites in the given class. Similarly, as presented in flux subsystem analysis, we used a “bootstrap without replacement” method to calculate *p*‐values and then adjusted these *p*‐values using the false discovery rate (FDR).

## CONFLICT OF INTEREST

The authors declare no conflict of interests.

## AUTHOR CONTRIBUTIONS

Q.P.K. conceived and designed the study. W.X., J.H., and G.L. designed the GPMM algorithm. G.L. developed the GPMM algorithm and performed modeling analysis. F.H. collected the benchmarking datasets. K.G., Q.S., and Y.W. collected samples and isolated RNA. W.X. and W.L. performed the statistical analyses. B.L. directed the metabolomics analysis. G.L., Q.P.K., W.X., and F.H. drafted the manuscript. G.L., Q.P.K., F.H.X., and F.H. revised the manuscript. All authors read and approved the final manuscript.

### OPEN RESEARCH BADGES

This article has been awarded Open Data and Open Materials Badges. All materials and data are publicly accessible via the Open Science Framework at https://github.com/GonghuaLi/Code_for_publications/tree/master/GPMM_Centenarians; https://github.com/GonghuaLi/GPMM.

## Supporting information

Fig S1‐S9

Table S1‐S7

## Data Availability

GPMM is available at https://github.com/GonghuaLi/GPMM. The codes for data processing and bioinformatic analysis are available at https://github.com/GonghuaLi/Code_for_publications/tree/master/GPMM_Centenarians. The CPLEX solver was used to conduct the flux balance analysis in this study. The RNA‐seq data generated for this paper have been submitted to the Genome Sequence Archive in BIG Data Center, Beijing Institute of Genomics, Chinese Academy of Sciences (GSA, http://bigd.big.ac.cn/gsa), under accession number CRA000515 (Xiao et al., 2018). The identified metabolic abundances are available in Table S7, and the metabolomic data have been deposited in the OMIX, China National Center for Bioinformation / Beijing Institute of Genomics, Chinese Academy of Sciences (https://ngdc.cncb.ac.cn/omix: accession no. OMIX733).
