# Supplementary material for: System‐level metabolic modeling facilitates unveiling metabolic signature in exceptional longevity
Source: Aging Cell. 2022 Mar 27;21(4):e13595. doi: 10.1111/acel.13595 (PMC9009231; doi:10.1111/acel.13595)
Supplement: Supplementary file 1 — Fig S1‐S9 [file ACEL-21-e13595-s001.docx]

**Supplemental Material**

This file contains:

Figures S1–S9


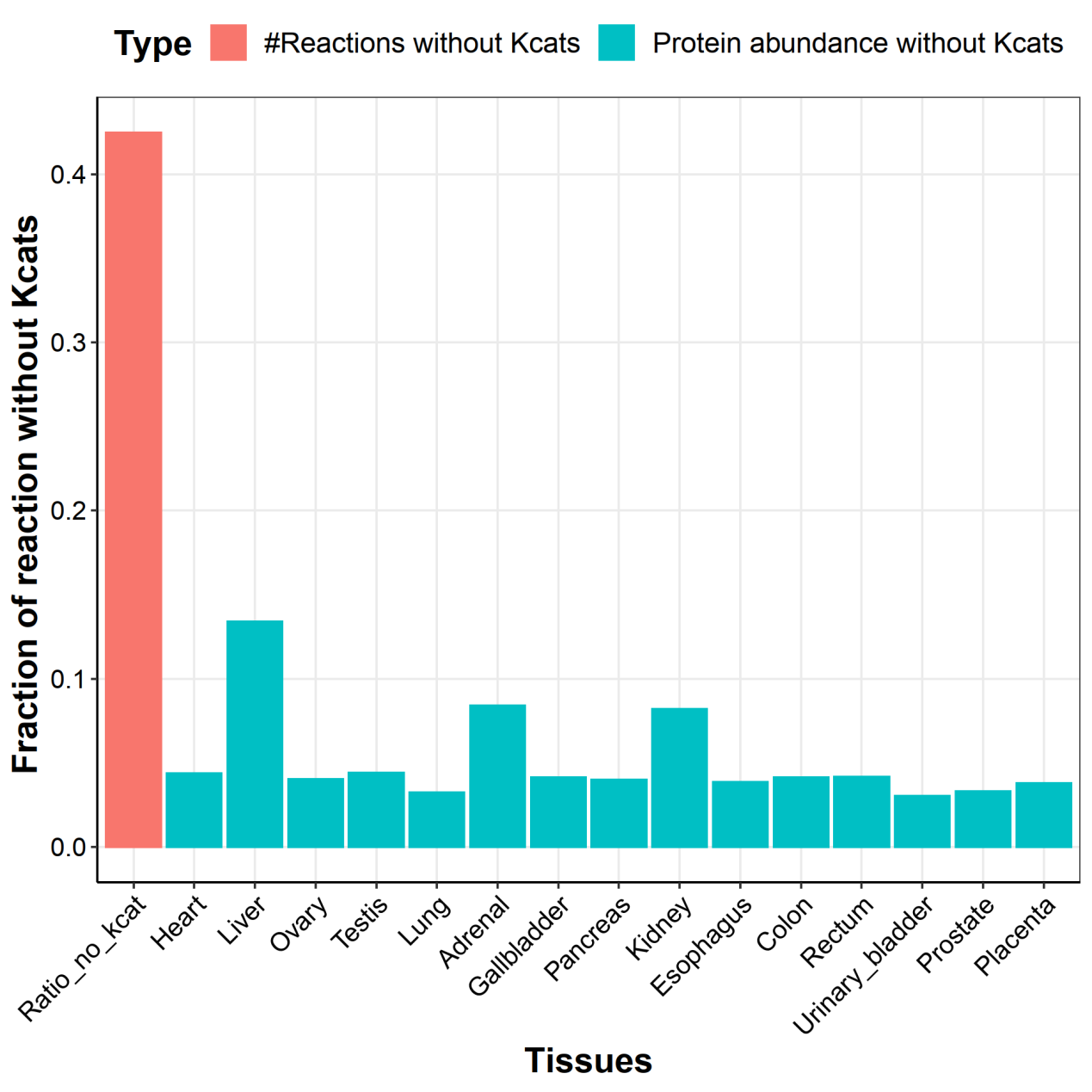


**Figure S1:** Enzyme abundance with unknown Kcat in Recon 3D in different tissues. Red: fraction of reaction without Kcats. Blue: fraction of protein abundance without Kcats.


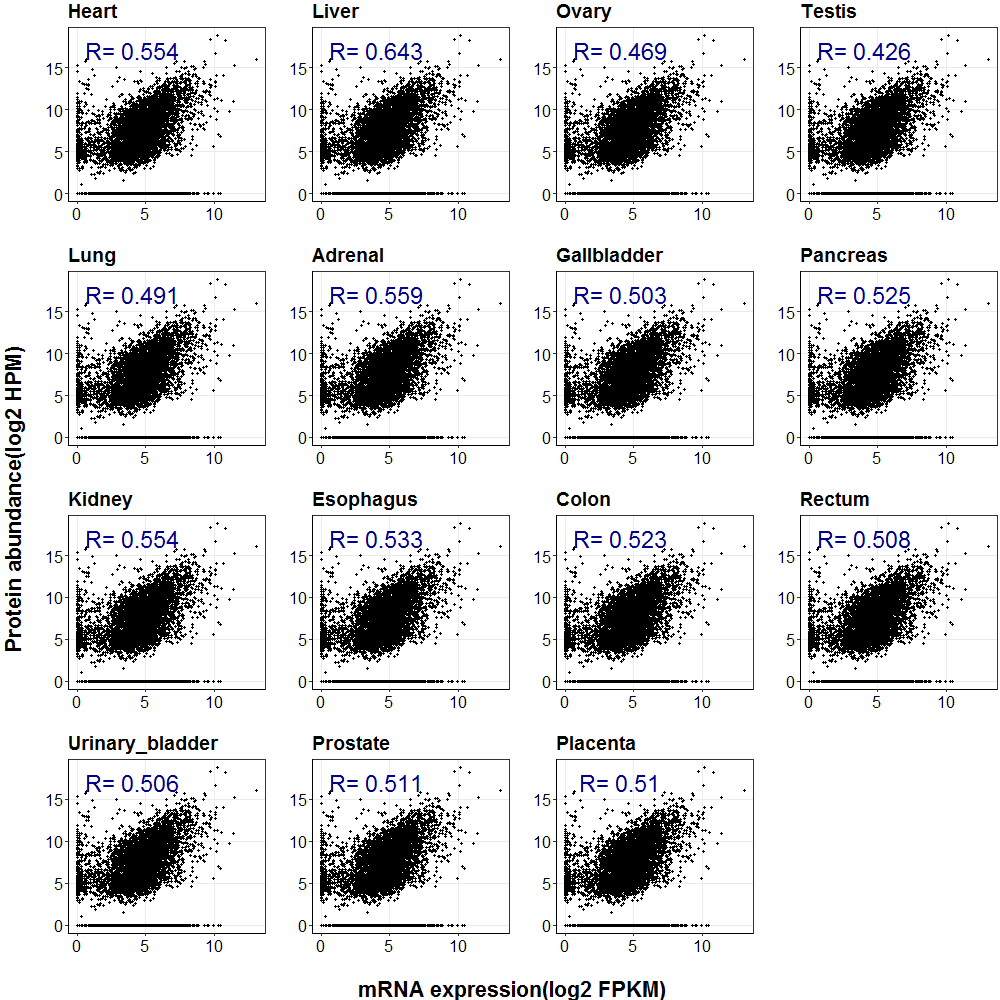


**Figure S2:** Correlation between entire mRNA expression level and protein abundance in different tissues. Protein abundance data are from draft map of human proteome. mRNA expression data are from human proteomics atlas (HPA).


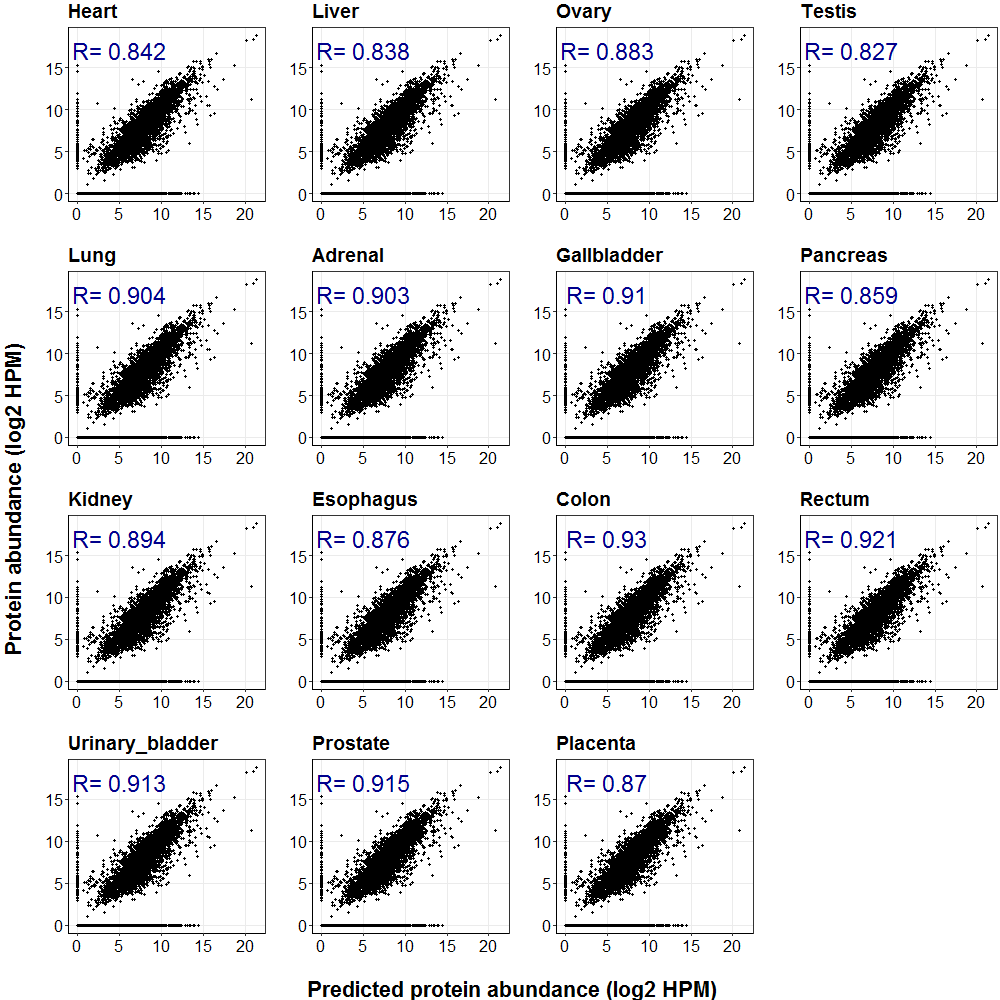


**Figure S3:** Correlation between predicted entire protein abundance and mRNA expression. Protein abundance data are from draft map of human proteome. mRNA expression data are from human proteomics atlas (HPA). Prediction was performed using mathematical model with equations 2–3.


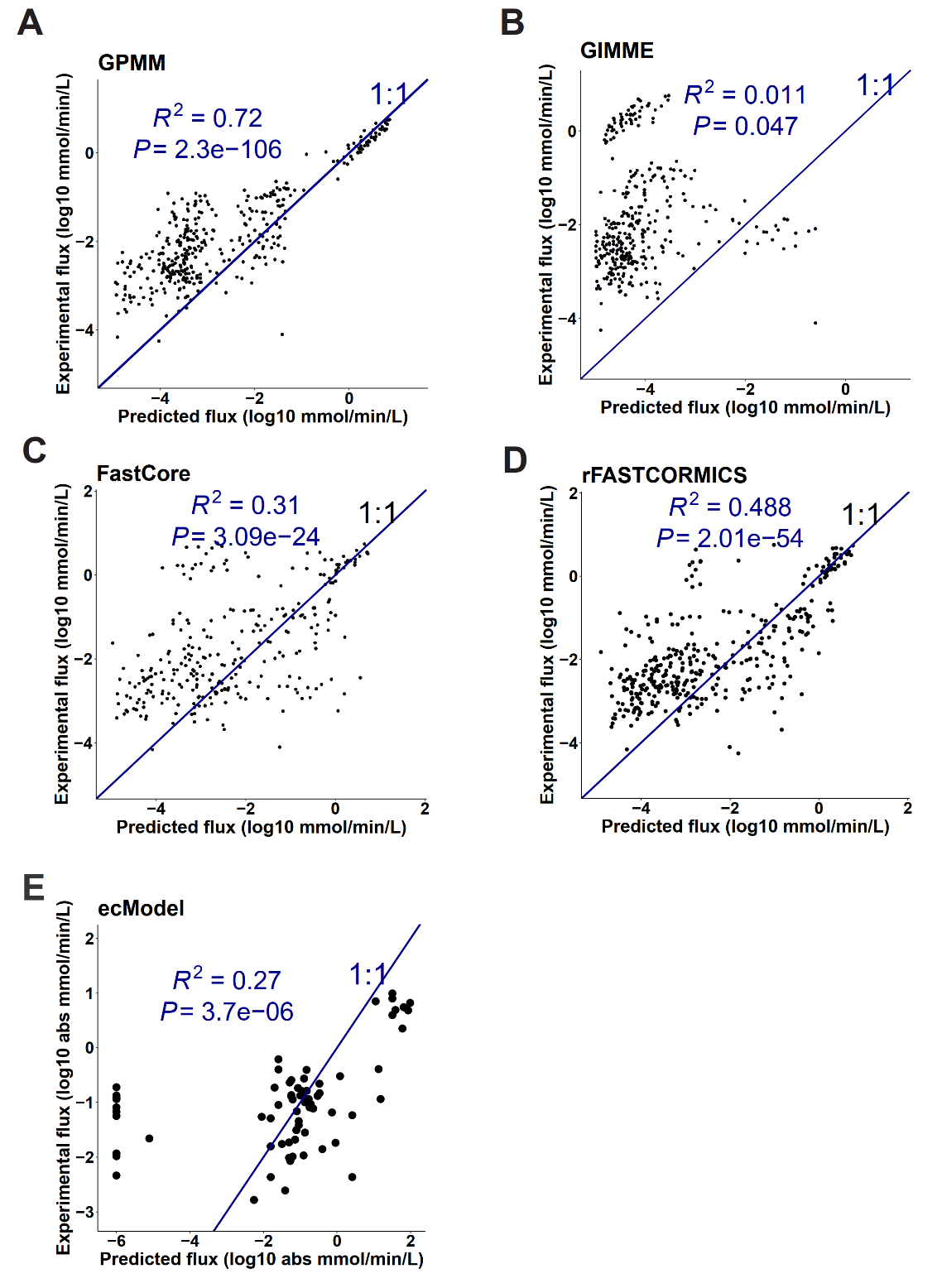


**Figure S4**: Comparisons between predicted metabolic fluxes and experimentally measurements in NCI-60 cells using GPMM (A), GIMME (B), Fastcore (C)， rFASTCORMICS (D) and ecModel (E) (R^2^ = 0.72, 0.011, and 0.31, 0.488, and 0.27 respectively). The predicted fluxes of ecModels are derived from Zenodo (<https://doi.org/10.5281/zenodo.3577466>) and only 11 ecModels are available.


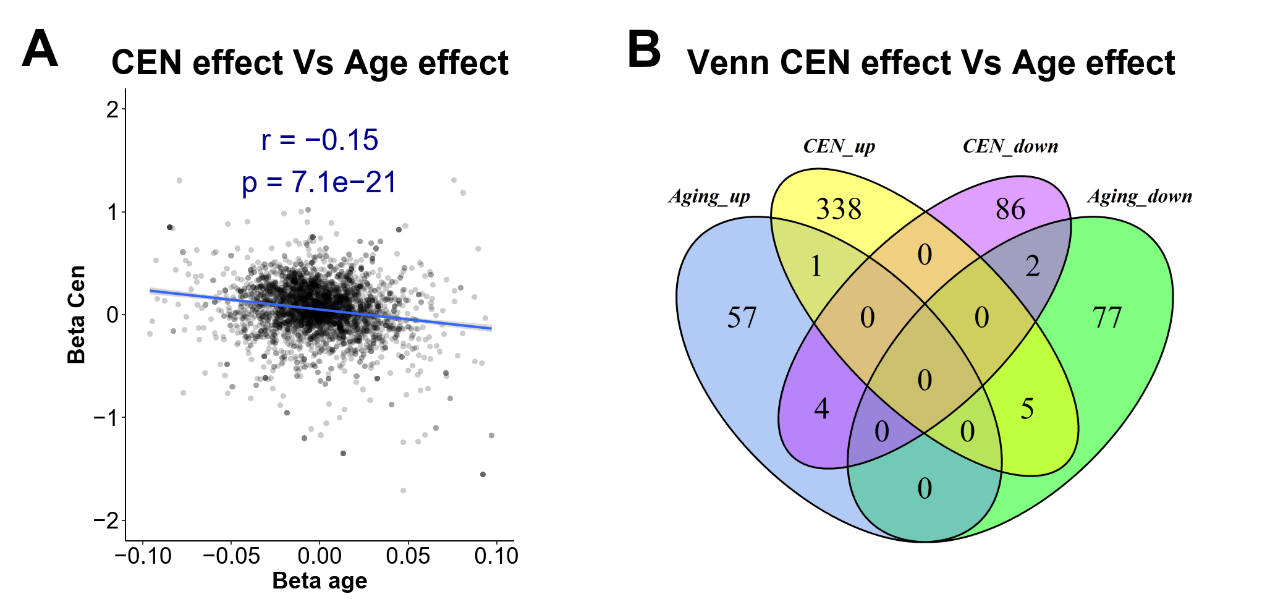


**Figure S5:** Comparison the overall centenarians and aging effect on fluxes. **A**: Overall relationship between the age effect and CEN effect on fluxes. Age and CENs has significantly negative correlation with the r = -0.15, *p* = 7.1e-21 **B**: Venn plot of the significantly changed fluxes in centenarian and the age effect.


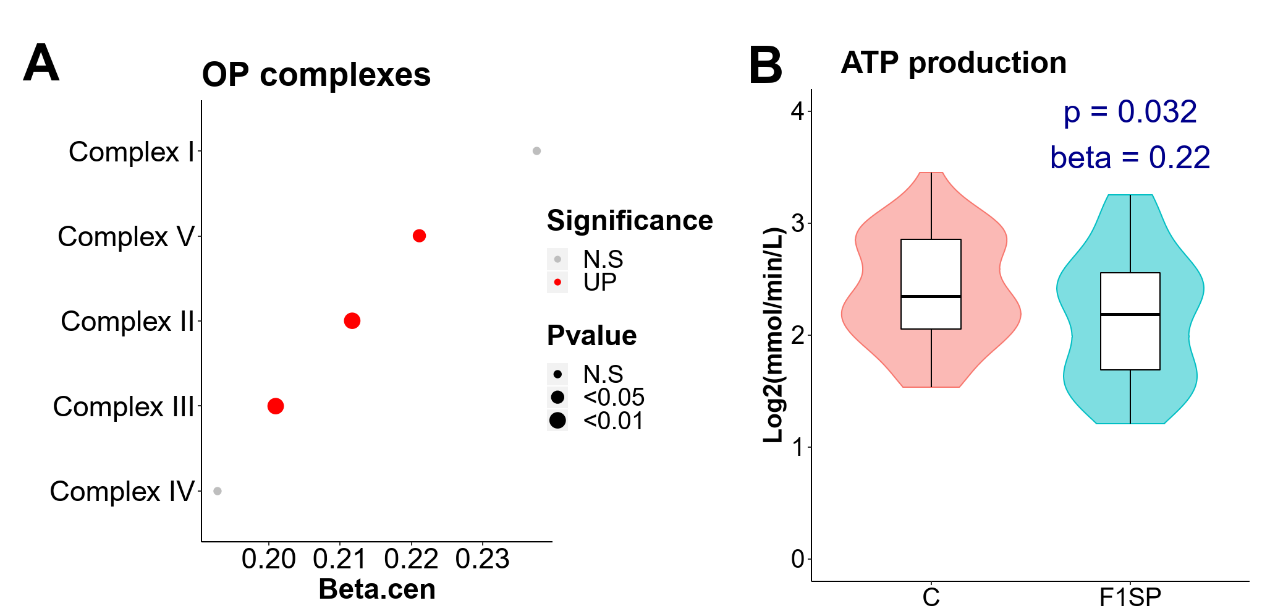


**Figure S6**: Flux changes in oxidative phosphorylation (OP) complexes (**A**) and adenosine triphosphate (ATP) production ability in centenarians (**B**).


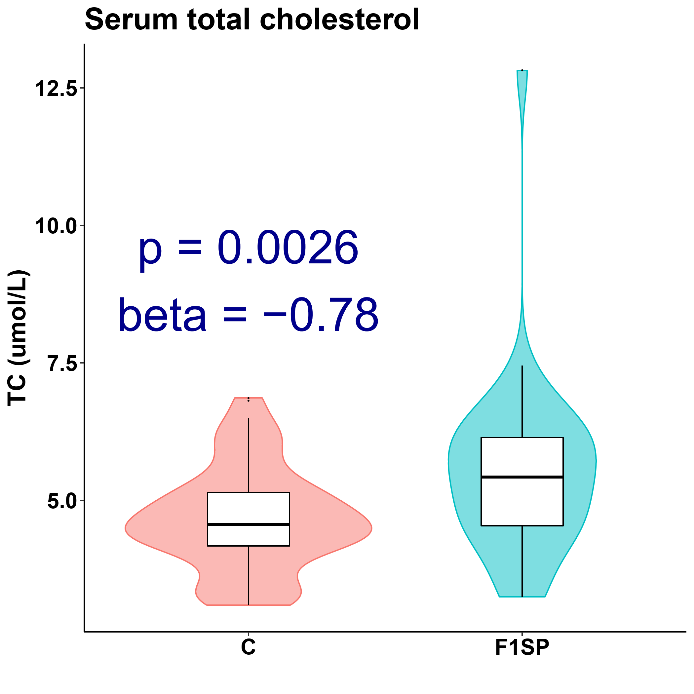


**Figure S7:** CENs showed lower total cholesterol (TC) abundance than F1SPs.


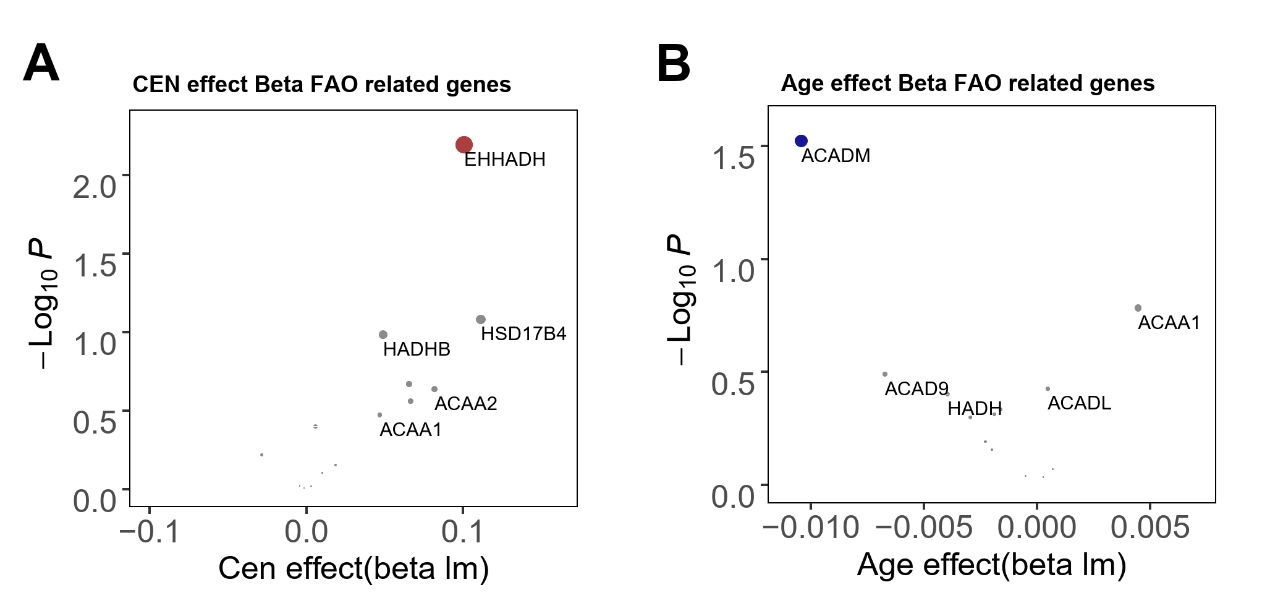
**Figure S8: Volcano plot of fatty acid beta oxidation related genes. A:** Centenarian effect of fatty acid beta oxidation related genes. **B:** Age effect of fatty acid beta oxidation related genes. **Note:** the fatty acid beta oxidation related genes are derived from Recon 3D. The significant upregulated and downregulated genes (p < 0.05) are colored by red and blue, respectively.


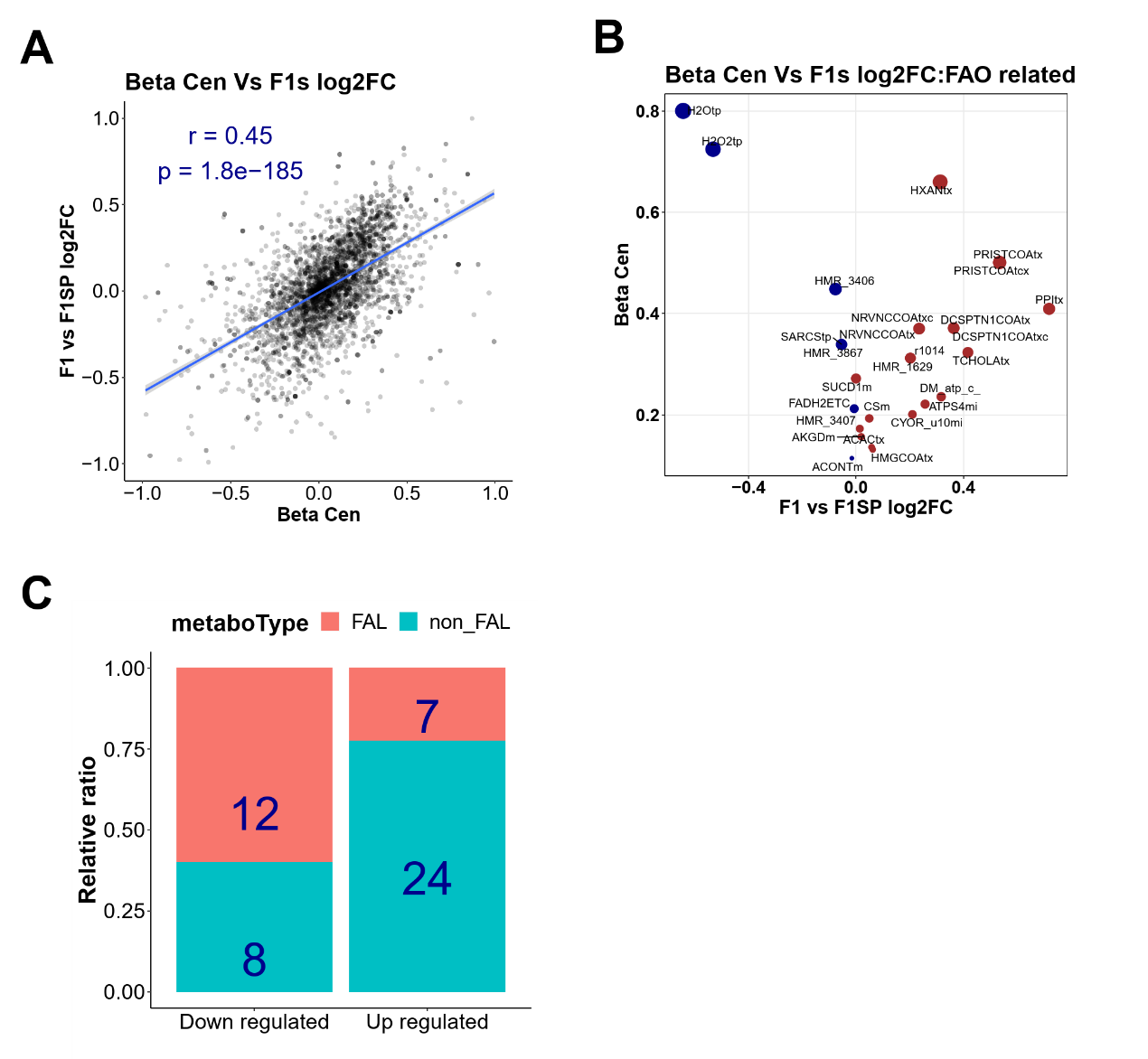


**Figure S9**: Comparison of F1s and Centenarian metabolic signature. **A**: Overall relationship between the F1s metabolic signature and CEN effect on fluxes. The overall CEN-specific flux signatures were significantly positively correlated with the Flux signatures in F1s (r = 0.45, p = 1.8e-185). **B:** F1s metabolic flux signature of CENs’ significantly upregulated FAO related reactions. Note: the FAO related reactions include fatty acid beta-oxidation, perisomal transport, citric acid oxidation, oxidative phosphorylation and ATP production. Purple and blue points represent log2FC>0 and log2FC < 0, respectively. **C:** Relative ratio of fatty acid-like (FAL) upregulated and downregulated serum metabolites in F1s.
